# Research ethics committees in a tight spot: Approving consent strategies for child research that are *prima facie* illegal but are ethical in terms of national guidelines

**DOI:** 10.7196/SAMJ.2018.v108i10.13203

**Published:** 2018-10-02

**Authors:** A E Strode, P P Singh, C M Slack, D R Wassenaar

**Affiliations:** 1 School of Law, College of Law and Management Studies, University of KwaZulu-Natal, Pietermaritzburg, South Africa; 2 HIV/AIDS Vaccines Ethics Group, School of Applied Human Sciences, College of Humanities, University of KwaZulu-Natal, Pietermaritzburg, South Africa

## Abstract

It is an internationally accepted principle that ethics norms should be applied and enforced in research with humans through ethics review by research ethics committees (RECs). This places RECs at the very heart of the system for protecting participants and enforcing their rights. In the South African ethical-legal framework for child research, there are divergent approaches to consent. That is, section 71 of the National Health Act (No. 61 of 2003) (NHA) requires mandatory parental consent for child research, and limits the authority for proxy consent to parents and legal guardians. However, national ethics guidelines authorised by section 72 of the NHA and issued by the National Health Research Ethics Council (NHREC) acting in terms of its mandate (National Department of Health, 2015) allow a more nuanced approach – i.e. self-consent by older adolescents, provided certain conditions are met, and consent by a range of parental substitutes where there are no available parents or legal guardians. We have argued elsewhere that the consent approach in section 71 is inappropriately restrictive and are of the view that the consent approach endorsed in national ethics guidelines is more defensible. An REC that elects to approve a consent strategy allowable in ethics guidelines is effectively electing to not follow section 71, which raises the question of what the consequences might be for that REC. This article examines the legal liability of RECs through three ‘threads’ of accountability: the NHREC, the institutions hosting RECs, and the courts. We conclude that: (*i*) if an REC approves a child protocol with consent strategies allowable in terms of national ethics guidelines but not in terms of section 71, it is unlikely that the NHREC would discipline the REC in the face of a complaint – provided the REC acted within national ethics guidelines issued by the NHREC in terms of the latter’s section 72 mandate to set national norms and standards; (*ii*) if an REC approves a consent approach allowed for in ethics guidance, it is also unlikely that the host institution would discipline the REC in the face of a complaint – especially if the institution is aware of the REC’s explicit decision to follow national ethics guidelines that are authorised by section 72 of the NHA; and (*iii*) an REC could only be sued by a participant in terms of the law of delict (and be liable for damages) if several demanding components are proven, such as that the harm suffered by the participant resulted directly from the REC’s actions in approving a particular consent strategy for that research. Furthermore, the court may well look to national ethics guidelines in making determinations about whether an REC’s conduct was wrongful for the purposes of liability in civil law. RECs are protected from being collectively liable by insurance taken out by their host institutions. We make a series of recommendations to address this issue.

For health research, law and ethics establish interlinked normative guidance. The legal system in South Africa (SA) is hierarchical – all law must be consistent with the Constitution of the Republic of South Africa, 1996.^[1]^ Furthermore, the norms established in laws override those that may be established in guidelines.^[2]^ Generally, ethics norms are not legally binding while legal norms can be enforced through the criminal or civil courts. Nevertheless, in certain circumstances ethics norms are indirectly enforceable through the courts, which use them as standards of acceptable behaviour when determining whether harmful conduct is actionable in terms of SA civil law.^[3]^ Despite ethics norms not being directly enforceable, there are nevertheless consequences for unethical decisions or practices, because it is an internationally accepted principle that ethics norms should be applied and enforced through ethics review undertaken by research ethics committees (RECs).^[4,5]^ RECs are at the very heart of the system of protecting and enforcing research participants’ rights.^[6]^

In SA, a deeply problematic aspect of the law, section 71 of the National Health Act (No. 61 of 2003)^[7]^ (hereafter NHA) requires mandatory parental consent for child research and restricts authority to provide proxy consent to parents and legal guardians.^[8–10]^ This approach may not be appropriate in all instances. For example, children may be involved in activities that make them vulnerable to parental disapproval or sanction (e.g. drug use), and parental consent for studies of such problems may place these children at risk of harm, or impede their enrolment.^[9]^ Children with no parents/guardians are precluded from research enrolment and such children as a class are precluded from research results that might benefit them.^[9]^ National ethics guidelines (National Department of Health (NDoH), 2015, 3.2.2.4)^[11]^ that are authorised by section 72 of the NHA allow a more nuanced approach to consent to child research which includes self-consent by older adolescents provided certain strict conditions are met. These guidelines allow for consent to be given by a range of parental substitutes where there are no available parents or legal guardians.^[8–11]^ It is important to note that the approach in SA national ethics guidelines is consistent with that in leading international ethics guidelines. More specifically, the Council for International Organizations of Medical Sciences (CIOMS) (2016)^[12]^ allows an REC to waive parental consent for child research when parental permission is not ‘feasible’ or ‘practicable’, when parental consent is not ‘desirable’, when studies investigate beliefs and behaviours where parental knowledge of the topic may place children at risk, and when the research poses no more than minimal risk.

We have argued elsewhere that the consent approach in section 71 is inappropriately restrictive, and are of the view that the consent approach endorsed in national ethics guidelines is more defensible and in children’s best interests. An REC that elects to approve a consent strategy allowable in national ethics guidelines is effectively electing to not follow section 71, which raises the question of what the consequences might be for RECs. What would be the implications if it was alleged that an REC has acted illegally but acted in accordance with national ethics guidelines? While there is some local literature on the liability of researchers or funders to pay compensation for enduring harm suffered by research participants, there seems to be very little on the liability of RECs in SA.^[13–18]^

This article addresses the question of what the consequences might be for RECs that approve *a consent strategy for child research allowable in national ethics guidelines but not in terms of section 71 of the NHA.* It examines the legal liability of RECs through three ‘threads’ of accountability – the National Health Research Ethics Council (NHREC), the institutions hosting RECs, and the courts. It illustrates these issues through two hypothetical adolescent protocols and discusses the legal implications that might follow from each. It concludes with recommendations on how to strengthen responses to this issue.

## Consent to child research in South Africa – divergent approaches

As set out in earlier articles, there are divergent approaches in SA law and national ethics guidelines regarding acceptable consent strategies for child research^[8–10]^ (a child is defined as a person aged <18 years). That is, in terms of section 71 of the NHA, a child cannot consent on his or her own to participate in health research.^[8–10]^ However, in terms of the national ethics guidelines^[11]^ there are circumstances when it may be ‘desirable’ and ‘ethically justifiable’ *for a minor to ‘choose independently’ (without parental assistance)* to take part in health research.^[8–11]^ These are when:

- The risks are minimal.^[11]^
- The child is ‘older’ (i.e. ≥16 years).^[11]^
- Researchers have provided ‘evidence’ of engagement with participating community role-players indicating that a waiver of parental consent is acceptable.^[11]^
- An REC has approved the approach.^[11]^

In addition, section 71 limits the persons with the authority to give proxy consent to parents and legal guardians.^[8–10]^ However, in terms of national ethics guidelines^[11]^ (which are authorised in terms of section 72 of the NHA), there are times *when alternative adults could provide proxy consent*.^[8–11]^ For example, the ethics guidelines allow (in section 3.2.2.3 entitled ‘Orphans without guardians’) that if there is no legal guardian, a *foster parent* could consent; if there is no foster parent, a *caregiver*; and if the minor is the caregiver in a child-headed household with no supervisory adult, a *trusted adult nominated by the minor*, including but not limited to a social worker, community worker or teacher.^[11]^ With specific regard to clinical trials, the NDoH (2006) Good Clinical Practice Guidelines (2.3.1.1) also assert that in some exceptional cases (e.g. ‘emergency’), caregiver consent for clinical trial enrolment is permitted,^[19,20]^ again illuminating the discrepancy between section 71 of the NHA and national guidance.

## The legal framework for the regulation of RECs in SA

Section 72 of the NHA established the NHREC, which in terms of section 6^[7]^ must *set norms and standards for conducting research on humans and animals*, including norms and standards for conducting clinical trials.^[7]^ The NHREC has issued norms and standards in the national ethics guidelines.^[11]^ These national ethics guidelines state that RECs are responsible for protecting the welfare and rights of participants.^[11]^ The ethics guidelines also describe the way in which ethics review should be done and stipulate that any decision taken by the REC ought to be recorded in writing.^[11]^ The recording of dissenting views of REC members is provided for.^[11]^ The guidelines do not, however, expressly state that a decision of the REC binds all its members. Nevertheless, it is submitted that this is implied within the broader content of the ethics guidelines.

Section 73 of the NHA creates an obligation on all ‘institutions, agencies or health establishments’ that conduct health research to ‘establish or have access to’ an REC registered with the NHREC.^[7]^ Section 73(2) of the NHA provides further that RECs are to:

- ‘review research proposals and protocols in order to ensure that research conducted by the relevant institution, agency or establishment will promote health, contribute to the prevention of communicable or non-communicable diseases or disability or result in cures for communicable or non-communicable diseases; and
- grant approval for research by the relevant institution, agency or establishment in instances *where research proposals and protocols meet the ethical standards* [authors’ emphasis] of that health research ethics committee.’

### Firstly, RECs are accountable to the NHREC.

This flows from the NHA, which obligates RECs to register with the NHREC. The NHREC is a national statutory body that undertakes both a normative and a regulatory role in research ethics.^[10]^ As above, the NHA in section 72(c) gives the NHREC the power to *issue ethics guidelines that are binding on all RECs*,^[7]^ and they can also audit RECs to ensure compliance with these national norms and standards.^[7]^ This allows the NHREC to hold RECs accountable by ensuring that they are administratively effective *and acting in accordance with the national ethics guidelines*. RECs that do not meet the ethical standards set by the NHREC could be de-registered and therefore be unable to continue to operate. This would leave their host institution without any means of granting ethics approval for research unless they were able to subcontract this task to an alternative REC. The NHREC may also take disciplinary steps against individuals who it has found to be *‘in violation of any norms and standards, or guidelines set for the conduct of research in terms of the Act’*.^[7]^ As above, acting in terms of its powers, the NHREC has duly published national ethics guidelines (NDoH, 2015),^[11]^ currently in their second edition.

### Secondly, RECs are accountable to the institutions that host them.

Host institutions can hold their REC accountable through institutional policies and disciplinary measures. In particular, they could act against their REC for bringing the institution into disrepute for failing to carry out their institutional mandate, and such action could include disbanding the current REC and appointing new members. Presumably the institution would have to show that the chair or a member acted in violation of the national ethics guidelines (and in contravention of standard operating procedures – a topic beyond the scope of this article).

### Lastly, RECs are accountable to protect the interests of participants.

This flows from SA civil law that allows a participant or their proxy consenter (a plaintiff) to bring a delictual claim, i.e. to bring an application for damages for harm they suffered as a result of the actions or omissions of the REC. It is conceivable that a civil claim might be brought against an REC that does not adhere to its legal duties. It is argued that this claim would be likely to arise against the REC itself as a juristic body and not its individual members. Some have submitted that REC members could be liable in their personal capacity where an REC acts negligently while exercising its review responsibility.^[18]^ However, we find this unlikely because: (*i*) individual REC members seldom, if ever, take official action on behalf of the REC without approval of the REC chair or indeed of the whole REC; (*ii*) at most institutions the REC chair is the responsible official who convenes and oversees REC decisions with authority mandated by the host institution; (*iii*) many RECs do not reveal the identity of reviewers of specific protocols and all correspondence is generally signed by the REC chair, in most cases anonymising individual REC members – so a legal complaint would have to address the REC chair or the host institution; and (*iv*) national ethics guidance requires that host institutions formally indemnify their REC members (including the chair) from legal action, suggesting that in the case of a legal complaint the host institution would be the respondent. Where an REC chair makes a decision alone and without consultation, as chairs are often required to do (e.g. on minimal-risk expedited review protocols), such decisions should be compliant with the NHREC-approved institutional terms of reference and/or the standard operating procedures of the REC in question. In such cases, the chair could not be held liable for such procedurally permitted decisions taken alone. Host institutions, in turn, should have public liability insurance to cover delictual claims. For this reason, the rest of this article addresses the responsibilities of the REC rather than its individual members.

If a participant (a plaintiff) wishes to pursue a claim for damages against an REC, he/she would have to prove:

- Firstly, an *act or omission*, i.e. the failure to apply section 71 of the NHA.
- Secondly, that the *act or omission is legally wrongful* because they failed in their duty to protect research participants. The NHA creates a duty of care on RECs towards participants. Should the REC fail to adhere to the requisite standard of care, it would have acted in a legally wrongful manner.
- That the act or omission (i.e. failure to apply section 71) was *intentional or negligent.*
- That the *wrongful conduct by the REC resulted in a legally recognised harm to the participant.* This could include either physical or emotional pain and suffering or some form of financial loss. It does not include emotions such as frustration, anger and disappointment.
- That there is a *causal link* between the conduct of the REC and the alleged harm.
- That the harm caused *cannot be legally justified* by the REC.

## Exploring the consequences for RECs through two cases

As set out above, section 71 of the NHA takes a highly restrictive approach to consent for child research by not allowing independent consent for research with persons aged <18, or non-parental proxy consenters, while the national ethics guidelines authorised in terms of section 72 of the NHA provide some flexibility and allow RECs to establish when independent consent or parental substitutes would be appropriate.^[8–10]^ It could be argued that RECs are legally obliged to apply the norms as set out in section 71 of the NHA even if inconsistent with ethics guidelines established in terms of section 72 of the Act by the NHREC. Given this background, what are the likely consequences for RECs that approve a consent approach endorsed in national ethics guidance but not in section 71? We test this issue in two hypothetical child studies that follow.

### Child study 1

A protocol is submitted to an REC. It proposes enrolling boys aged 16 – 17 years who identify as MSM (men who have sex with men) or who describe same-sex practices. The protocol identifies this group as especially at risk of HIV infection and stigma or discrimination, and describes how inadequate knowledge about their preferences for services is a serious impediment to the design of tailored services. The study aims to explore risk behaviour in this group, and to explore their stated preferences for risk reduction services. Study methods will include in-depth interviews and surveys. The protocol states that early engagement with a community advisory board and conversations with boys representing the same demographics as the proposed sample indicate that parental consent will deter boys from enrolment because in most instances parents are not aware of such socially stigmatised behaviour. In many instances parents might react with disapproval or sanctions – the latter would constitute social harms from the study that cannot be reduced to an acceptable minimum. The protocol proposes adopting an independent consent strategy, with optional ‘opt in’ of parental involvement should this be desired by the participant (whereby site staff would meet with parents and discuss the study in the presence of the participant/with their permission). The REC regards the risk of the study procedures (interviews and surveys) – with risk mitigation strategies such as anonymisation – to be ‘minimal’ (commensurate with routine medical and psychological tests and exams). The REC approves the protocol, with an independent consent approach, ensuring that all foreseeable risks, including stigma and discrimination, are outlined in the information sheet at an age-appropriate reading level. A 17-year-old boy enrols in the study, providing independent consent. The boy’s father subsequently discovers his son’s participation in the study, infers that his child is engaged in same-sex practices, and severely assaults the child. The assault is reported as an adverse event to the REC. The father alleges that the REC should be held accountable. What are the possible consequences for the REC in this scenario?

In terms of the *first layer of accountabilit*y through the NHREC, it is submitted that the REC has acted justifiably as they followed the NDoH (2015) ethics guidelines^[11]^ issued by the NHREC. It is therefore unlikely that the NHREC would take any action against the REC.

In terms of the *second layer of accountability*, it is unlikely that an institution would take action against their REC in this instance, given that the REC’s decision was ethically justifiable according to national ethics guidelines (NDoH, 2015).^[11]^

In terms of the *third layer of accountability* (delictual liability), it might be argued that the act was that of the REC approving a self-consent strategy for this study, that this was wrongful as it was contrary to the legal obligation to obtain mandatory parental consent in terms of section 71 of the NHA, and that it occurred intentionally because the REC made a conscious choice to follow national ethics guidance rather than the NHA. The harm that resulted from this wrongful, intentional act was parental ignorance of their child’s enrolment and the subsequent assault. There is a clear link between the consent approach and parental ignorance of study participation; however, it is less obvious whether a clear link could be shown between the consent approach and the harm (i.e. the assault). It appears that the harm (the assault) was more likely to have directly resulted from pre-existing parental prejudice against the adolescent’s sexual orientation than from study enrolment. Also, it is possible that the court would not regard the REC’s actions as ones that ought to result in legal liability because they were not legally wrongful. It is possible that the court might hold that the legal convictions of the community would not deem conduct in accordance with the ethics guidelines to be legally wrongful as such conduct is in the best interests of the child participant and is clearly ethical conduct in terms of the national ethics guidelines.^[11]^ We submit that the outcome for the REC would be the same even if the complainant was the adolescent participant himself. (This scenario assumes that the consent approach approved by the REC did not involve coercive elements, where site staff would express or imply that adolescents refusing enrolment would be worse off in future, e.g. receive fewer or lower-quality services.)

### Child study 2

A protocol is submitted to an REC. It proposes enrolling children of 10 years and older from child-headed households. The study aims to explore their experiences of living without parents or guardians, and their use (or not) of relevant support services. The protocol identifies this group as especially vulnerable to ‘falling between the gaps’ in existing services. Study methods will include in-depth interviews with some potentially distressing questions. The protocol states that parental consent is impossible as per section 71 because such participants have neither a parent nor a legal guardian. The protocol proposes obtaining consent from a local priest operating several child-related services in the area as well as the assent of each child. The REC regards the risk of the study procedures (interviews) – with risk mitigation strategies such as anonymisation – to be ‘minimal’ (commensurate with routine medical and psychological tests and exams). The REC approves the protocol, including the consent approach. Later, an enrolled child tells her caregiver that taking part in the study procedure (the interview) was distressing for her, and the caregiver complains that the child suffered harm from the distressing questions, and that she (as the caregiver) would not have approved the child’s enrolment had her permission been sought.

In this scenario, in terms of the *first layer of accountability* through the NHREC, the NHREC could take action against the REC for failing to adhere to national ethics norms that provide explicit direction regarding acceptable parental substitutes, where research involves orphaned or vulnerable children.^[11]^ However, given the relatively minor nature of the ‘harm’ in this scenario, it is assumed that steps might be taken to resolve the matter informally. In terms of the second layer of accountability, it is also possible that an institution could take action against their REC in this instance, given that the REC’s decision to approve the consent approach was not consistent with national ethics guidance.

In terms of the *third layer of accountability* (through the civil law), it could be argued that the act was that of the REC approving this consent approach, that this was wrongful as the consent strategy was not allowable in terms of section 71 of the NHA, and that this was an intentional act as it can be assumed that the REC deliberated on it. The harm that resulted from this wrongful, intentional act was emotional distress from the interview. In this case it appears that there is a clear link between the consent approach and the harm (emotional distress), because a young child was exposed to personal questions without the involvement of her caregiver. Also, it is likely that society would regard the REC’s actions as ones that ought to result in legal liability because the REC did not act in accordance with national ethics guidelines. Further, our law of delict recognises a claim for psychological harm where such harm is not of short duration, and the child would be entitled to damages if she could prove long-term psychological harm. This amount, however, would be minimal and only cover the costs of receiving psychological counselling for her emotional distress.

## Conclusions

RECs have been placed in a tight spot when it comes to approving child consent approaches that are inconsistent with section 71 but are consistent with national ethics norms and also with long-established and current international ethical norms (such as CIOMS, 2016^[12]^).

RECs are faced with having to make a conscious choice between acting ‘ethically’ or ‘legally’. We argue that they ought to act in terms of their core mandate to apply the national ethics guidance and act ethically, as to do so is in the best interests of children.

Nevertheless, this choice – to approve a child protocol with consent strategies allowable in terms of national ethics guidelines,^[11]^ but not in terms of section 71 of the NHA – may have consequences for an REC. Firstly, a complaint may be made to the NHREC. However, it is unlikely that the NHREC would discipline the REC if it acted in accordance with national ethics guidelines issued by the NHREC in terms of the latter’s section 72 mandate to set national norms and standards. Secondly, it is possible that the host institution could discipline the REC. Again we see this as unlikely, especially if the institution is made aware of the REC’s decision to follow national ethics guidelines rather than the restrictive legal approach. Thirdly, an REC could be sued in terms of the law of delict. However, s*everal demanding components would have to be proved*, such as that harm suffered by a participant resulted directly (or was causally linked) to the REC’s approval of a non-parental consent strategy. This is a very demanding approach, as our hypothetical cases illustrate. In addition, it is likely that our courts will hold the REC’s approval not to be legally wrongful if they conclude that the legal convictions of society would approve of the REC acting ethically rather than legally, especially in situations where such conduct is in the best interests of children, rather than strictly adhering to the restrictive requirements of section 71 of the NHA.

### Recommendations

RECs should only approve consent strategies that deviate from the consent approach set out in section 71 where this approach is clearly provided for in national ethics guidance. If RECs are to follow national ethics guidance, they should make a conscious choice to do so and should document their decisions carefully to show that they have made their decision based on the norms and standards of national ethics guidelines. This may also assist them if there was an NHREC audit to assess whether national ethics norms were being followed. RECs should advise researchers to use criteria set out in the national ethics guidance to ethically justify any waiver of parental consent in their protocol submissions. RECs should also inform their institutions about their decision to follow the guidelines. Until such time as the NHA (2003)^[7]^ and NDoH (2015)^[11]^ are aligned, the NHREC should provide guidance on how to approach this tension. Institutions should develop policy positions on this issue given that they could be delictually liable if the demanding test could be met that the harm suffered by a participant was causally linked to the REC’s decision to approve a particular consent approach. The internal contradiction between section 71 and section 72 (and section 73) requires urgent attention from policy-makers to ease the pressure for RECs attempting to facilitate socially valuable research for children.
